# Spatial distribution patterns and factors influencing rural tourism destinations: An empirical study of China’s agritainment resorts

**DOI:** 10.1371/journal.pone.0308415

**Published:** 2024-09-12

**Authors:** Lei Zhu, Jing Hu, Jiahui Xu, Yannan Li, Tiantian Xie, Mangmang Liang

**Affiliations:** 1 School of Resources and Environment, Anqing Normal University, Anqing, China; 2 College of Urban and Environmental Sciences, Central China Normal University, Wuhan, China; Politecnico de Milano, SPAIN

## Abstract

Agritainment is one of the essential aspects of rural tourism and plays an important role in the economic transformation and revitalization of rural areas. Taking 9200 agritainment resorts in China as a research object, this paper systematically uses geospatial analysis methods to analyze their spatial distribution patterns and influencing mechanisms. The results indicate: (1) All types of agritainment have a condensed distribution in space and are oriented in the northeast—southwest direction, with a central axis generally located in the Beijing–Zhengzhou–Wuhan line. (2) The distribution of agritainment is uneven across different spatial scales, and there are high-density clusters in the Beijing–Tianjin–Hebei region, the Yangtze River Delta, and the Sichuan–Chongqing region as the core, and sub-high-density distribution areas in the Shaanxi–Gansu–Ningxia border, the southern coastal region, and the Xiangan–Jiang–Hubei border, manifesting prominent spatial distribution characteristics of large agglomeration and low dispersion. (3) Agritainment has a significant positive spatial autocorrelation. The Matthew effect is highly significant in space. The distribution of cold hot spots in the agritainment space shows a distribution pattern of "hot in the south and cold in the north." (4) The spatial distribution of agritainment is influenced by human factors such as society, economy, and the tourism industry as well as natural factors such as terrain, water systems, and climate. The intensity of influence of first-level human factors on the spatial distribution of agritainment ranks as follows: tourism industry factors (0.69) > social factors (0.37) > economic factors (0.30). The natural distribution of agritainment tends to be in humid plain and hilly areas with an altitude below 1000 m and annual precipitation above 800 mm. Agritainment is mainly distributed in the subtropical monsoon climate area adjacent to rivers. The research findings offer valuable insights for optimizing the spatial distribution pattern of agritainment in China, promoting the high-quality development of agritainment, and the sustainable development of rural tourism.

## 1. Introduction

Rapid socio-economic development is exerting growing pressure on the life and work of urban residents; consequently, increasing numbers of urban residents flee the city to the countryside to relax and experience rural life. Therefore, in recent years, rural tourism has become highly sought after by tourists. As one of the most important forms of rural tourism, agritainment spaces have increased rapidly in all countries in the world. Agritainment is positioned outside a city or town limits and close to the countryside and is created by both farmers and city dwellers [1]. Agritainment spaces are not a venue to sell products or crafts and often have a vast space with a yard. People pay money to travel there not to shop but rather to talk, eat, drink, sing, do business, and play mahjong and cards [2]. Agritainment provides amusement for visitors through its offerings, namely, venues, tea houses, regional foods, rural accents, and picturesque settings [3]. Consequently, agritainment is a genuinely unique recreational and shopping location that may be found in China’s modern rural—urban border areas.

Since 2000, entertainment along with consumer culture and the rapidly growing fashion scene have spread throughout China. This phenomenon is distinctive not only because it is rarely found elsewhere but also because of its pronounced Chinese traits [4, 5]. Chinese people support and love public consumer places; moreover, the government endorses and promotes these places. Although Chinese consumer culture is growing, it is still uncommon for the elite and lower classes to share a common appreciation [6]. This condition may be viewed as the “aestheticization of everyday life” or a Chinese way of enjoying life [7]. We seek to obtain an understanding of the most distinctive characteristics of Chinese cultural and consumer space via the interpretation and analysis of 9200 agritainment spaces. A multilayered public consumer space network for entertainment has already been built in most of the large Chinese cities [8]. By the end of 2020, there were over 2 million attractions, which welcomed 50% of China’s population, or approximately 700 million visitors annually, according to official figures.

Agritourism may be divided into rural agritourism, which is operated by local farmers, and large-scale agritourism, which is controlled by family enterprises and features specialization and professionalization [9]. Agritainment establishments feature various sorts of faux antiques, fabricated artificial landscapes, and a farmhouse ambiance. The price per person per day might be as low as 20 or 30 yuan or as much as hundreds of yuan when experiencing a variety of activities [10, 11]. Both common people and upper classes engage in agritainment to fulfill their consumption needs. This phenomenon is particularly common in the Zhejiang and Jiangsu regions. The Zhejiang and Jiangsu governments are attempting to create the most alluring “leisure city” in China, and the entertainment industry is one of the key facets of a modern Chinese “leisure culture” set to be promoted and developed [12, 13]. Especially after the COVID-19 pandemic, farmhouse tourism has attracted increasing attention from various groups of people.

From a consumerist perspective, entertainment is merely a commercial endeavor. We can classify consumption, entertainment, and leisure under the umbrella term “agritainment,” which is defined by Geng et al., as overtly “seeking fun from everyday life” [14]. Agritainment is the product of the valorization of everyday knowledge and life skills that can be directly transformed into material goods or the pleasures of a holiday [15]. The “aestheticization of everyday life” based on “the project of turning life into a work of art” has emerged as a distinct consumer culture in the interim [16]. However, in contrast to Western consumer culture, people who support agritainment—or the “aesthetic presentation of everyday life”—are neither artists nor thinkers [17]. The majority of agritainment inputs come from neighborhood farmers who are from a low social class yet are aware of business and economic operations. They were then joined by other social actors (businesspeople, individual artists, etc.), and eventually, agritainment caught the attention of the authorities, who earnestly advocated it.

More specifically, most research focuses on the financial advantages of agritainment [18]. It has been shown that improving farm products, expanding market accessibility, and diversifying the conventional farm enterprises’ revenue streams are the key ways to boost income [19, 20]. Since yields and prices are not entirely controllable over time, agritainment has made significant progress in addressing the production, pricing, technical, and political concerns of extremely variable agricultural revenue [21]. The potential for the rural eco-tourism sector to boost local production, earn foreign cash, draw investments, and create new employment as an engine of economic growth has made it increasingly popular and helpful for the development of the local economy [22]. It also discusses the critical role that agritainment plays in non-economic advantages including promoting social justice, environmental sustainability, rural cultural revitalization, and heritage preservation [23].

Scholarly research on agri-tourism mainly focuses on different types of agri-tourism business models, tourism product suggestions, and community participation [3, 24, 25]. There are few studies on the spatial layout of Chinese agritainment. Nevertheless, although the emergence of geospatial big data has brought great opportunities for research from a spatial perspective, the spatial characteristics of agritainment are still under-studied. At present, scholars have only made a simple analysis of the spatial distribution of agritainment in some provinces of China. The whole research method is relatively simple, the research depth needs to be improved, and there is a lack of macroscopic systematic research on the national scale. In particular, studies on the influencing factors of the spatial distribution characteristics of agritainment tend to focus only on social factors, and lack of studies on physical geographical elements. To fill this research gap, in this study, a systematic geospatial analysis method was used to systematically explore the spatial distribution characteristics of 9200 agritainment in China, and on this basis, an influence system with two dimensions of humanity and nature was constructed to comprehensively explore the influence mechanism of their spatial distribution. On this basis, the development pattern and path of Chinese agritainment are proposed, with a view to providing ideas for the identification, evaluation, and rational development and construction of rural tourism sites in the future. It can also be used to optimise the spatial layout pattern of Chinese agritainment, promote the construction of beautiful villages and the high-quality development of rural tourism, enrich the content of agro-geographical research, and have a certain reference value for the formulation of agricultural policies at the national level and in different regions.

## 2. Literature review

### 2.1. Progress of rural tourism research

Rural tourism was seen by industrialized nations prior to the 1990s as a key means of transforming the rural economic structure in an effort to overcome the challenge of rural decline. Thus, rural tourist operators’ primary goals now include managing farm crises, achieving agricultural diversification, subsidizing agricultural revenue, and pursuing financial interests [26]. Business motivation has exhibited diversification, encompassing various areas such as economy, social culture, and politics, after the mid-1990s, when the growth of rural economies and the improvement of living standards undermined the dominance of economic drive. It is noteworthy that in an Irish hamlet, just 1.7% of the residents engage in rural tourism for profit.

Socio-cultural motivation is primarily influenced by rural living, family dynamics, social contact, and autonomous work. For instance, Thompson’s research of a Japanese village demonstrates that the primary drivers of rural tourism among the local populace are affirming the significance of the contemporary rural lifestyle, preserving local customs, and strengthening local identity [27]. The primary drivers of middle-aged couples operating rural tourism are their preference for a rural lifestyle and social issues relating to family, according to a Gets et al. survey done in rural western Australia [28].

Through a case study, Paniagua came to the conclusion that Spanish citizens who followed the counter-urbanization trend and relocated to rural regions mostly engaged in rural tourism in order to become self-employed [29]. Today’s globalized capitalist economy and shifting global ideologies have led to the emergence of a new, service-industry-based polity with a local government structure. Thus, from the standpoint of political economy, the local government’s decision to adjust policies to the changing political climate is what drives the growth of rural tourism [30].

### 2.2. Progress of agritainment research

Agritainment is an important carrier of leisure agriculture and an important form of rural tourism development, which has gradually attracted the attention of scholars in rural tourism research. Scholars carry out systematic research on agritainment mainly from four aspects [31].

The first is the study of local residents’ perception and attitude towards agritainment. Long found that residents’ attitudes towards agritainment changed with the different stages of agritainment development. In the initial stage, most residents are in favor of the development of agritainment, but when the number of tourists reaches and exceeds the maximum social carrying capacity of the local population, the support of local residents will decline [32].

The second is to study the main body of agritainment management. Scholars pay more attention to the study of the main body of agritainment management, including: population characteristics, business motivation, business willingness and business performance. Bagi found that compared with traditional agriculture, farm owners had higher education levels and were better at using information technology [33]. McGehee analyzed the performance of 331 farmers in Virginia and found that 42.8% of the farmers’ annual household income was between $50,000 and $100,000, 23.2% of the farmers’ annual household income was more than $100,000, and 33.9% of the farmers’ annual household income was less than $50,000 [34].

The third is the study of the influence of agritainment. The development of agritainment has a certain impact on the local economy, social culture and environment. Akpinar believes that agritainment can not only promote the diversification of agricultural development modes, provide more market sales opportunities for agricultural products, but also help create more employment opportunities and increase the income level of local residents [35]. Foreign scholars believe that the sociocultural influence of leisure agriculture has both positive and negative aspects [36].

The fourth is the study of the spatial structure of agritainment. With the rapid development of leisure agriculture, the study on the spatial distribution and causes of leisure agriculture has become a hot issue. Scholars systematically used spatial analysis methods (nearest neighbor index, nuclear density, disequilibrium index, exploratory spatial data analysis, etc.) to study the spatial distribution of rural tourism destinations and leisure agriculture in China and the United States. The influence mechanism of the spatial distribution of rural tourism destinations is explored by using the spatial measurement research methods such as geographic detector, space superposition and buffer analysis, and qualitative analysis [37]. However, as a study on the spatial structure of agritainment, only scholars have studied its spatial distribution and influencing factors from the whole province or regional scale, and there is a lack of large-scale research in the country [38]. In addition, the research on the influencing factors of spatial distribution is also relatively weak, and the relevant influencing factors are not selected from the two dimensions of nature and humanity to systematically explore the influencing mechanism of the spatial distribution of agritainment.

It is not difficult to find that scholars have a wide range of research contents on rural tourism, and the results of studying the spatial structure of rural tourism destinations with different objects as the carrier are increasingly rich. However, as a typical spatial unit and carrier of rural tourism destination, the research results on its spatial structure are relatively weak. In view of this, this study takes 9,200 gold medal agritainment resorts with the highest level in China as the research object, and comprehensively uses GIS spatial analysis techniques such as nearest proximity index, nuclear density index and disequilibrium index to systematically explore the spatial distribution law of agritainment in China. The main factors of the spatial distribution of Chinese agritainment were identified and their formation mechanism was analyzed by means of polydetector, spatial superposition and buffer analysis. On this basis, the development model and path of China’s agritainment are proposed, in order to provide ideas for the identification, evaluation and reasonable development and construction of rural tourism destinations in the future.

## 3. Materials and methods

### 3.1. Data sources

**Fig 1:**
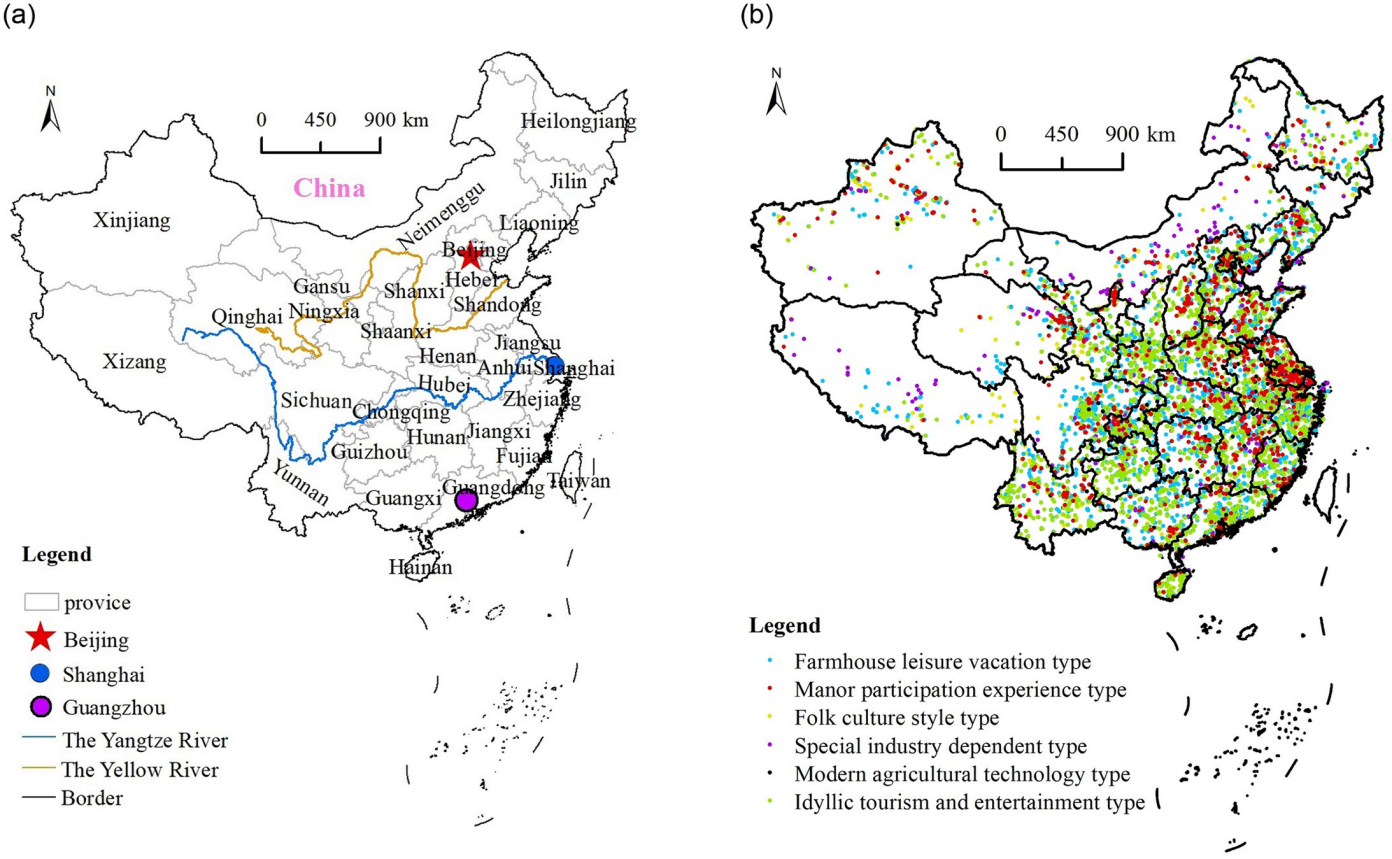
Spatial distribution of national agritainment. Reprinted background map from the National Catalogue Service for Geographic Information (www.webmap.cn) under a CC BY license, with permission from the Ministry of Natural Resources of China, original copyright 2023.

The study area covered 31 provinces, municipalities, and autonomous regions in mainland China, excluding agritainment resorts in Hong Kong, Macao, and Taiwan. 9200 gold medal agritainment resorts date were obtained from the official website of the Ministry of Culture and Tourism (https://www.mct.gov.cn/; accessed on 25 May 2022). These resorts are of the highest standard in China and exemplify exemplary development. These agritainment resorts can be divided into six categories (Table 1): modern agricultural technology (128 resorts), farmhouse leisure vacation (3625 resorts), manor participation experience (940 resorts), idyllic tourism and entertainment (3558 resorts), special industry-dependent (551 resorts), and folk culture style (398 resorts). We used Google Earth to determine the precise coordinates of these resorts and utilized ArcGIS 10.2 software to visualize and map the spatial distribution of agritainment in China (Fig 1). On this basis, the spatial distribution patterns and factors influencing the development of agritainment resorts were analyzed and studied. The selection of the influencing factor index was based on the feasibility and availability of data, with a focus on the two aspects of humanity and nature. The index system of human factors was mainly constructed from three dimensions: economy, society, and the tourism industry; and the relevant index data was obtained from the official website of the National Bureau of Statistics (http://www.stats.gov.cn/; accessed on 25 May 2022). Moreover, natural factors were systematically explored based on terrain, precipitation, rivers, and climate. The national vector map of 1:4 million in the national basic geographic information system database was used as a source of basic vector data in this study. The data in China’s DEM digital elevation (resolution of 30 m), precipitation, river system, and climate division were all derived from the Resources and Environmental Science and Data Center, Institute of Geographic Sciences and Natural Resources Research, Chinese Academy of Sciences (http://www.resdc.cn; accessed on 25 May 2022).

**Table 1:** Ellipse parameters of the nearest neighbor index and standard deviations of various types of agritainment in China.

Type	Nearest Neighbor Index	Distribution Type	Longitude of center point /°E	Latitude of center point /°N	Long half shaft Length /km	Short half shaft Length /km	Corner θ/(°)
Modern agricultural technology type	0.484	Remarkable aggregation	115.29	32.62	828.98	1038.29	15.51
Farmhouse leisure vacation type	0.337	Remarkable aggregation	112.26	33.22	941.76	1070.19	41.92
Manor participation experience type	0.470	Remarkable aggregation	112.75	33.83	1098.58	983.11	119.31
Idyllic tourism and entertainment type	0.367	Remarkable aggregation	112.43	31.99	900.57	1056.31	28.73
Special industry dependent type	0.489	Remarkable aggregation	112.75	34.38	1135.41	1242.24	56.87
Folk culture style type	0.450	Remarkable aggregation	103.34	37.17	1553.79	1275.56	97.80
Total	0.236	Remarkable aggregation	112.06	33.06	1024.16	1085.43	45.07

### 3.2. Study methods

#### 3.2.1 Nearest neighbor index

The nearest distance is a geographical index that reflects the degree of proximity of point-like objects in geographic space. Point elements mainly include the three spatial distribution states of agglomeration, uniformity, and randomness [39]. In this study, the method of the nearest neighbor index was used to identify the spatial distribution type of agritainment. The calculation formula is

$$R=\frac{\bar{r_{1}}}{\bar{r_{E}}}$$ (1)

where ${\bar{r}}_{E}=\frac{1}{2\sqrt{D}}=\frac{1}{2\sqrt{n/A}}$ is the theoretical value of nearest distance, is the average actual value of the nearest distance, A is the area of the study site, n denotes the number of agritainment resorts, and D is the density of the study site, which indicates the proximity of agritainment in geographic space. *R* = 1 represents a random state distribution. *R* > 1 represents a uniform state distribution, while *R* < 1 represents a cohesive state distribution.

#### 3.2.2. Kernel density analysis

The results of kernel density analysis can reflect the influence of core agglomerations on surrounding areas, and high values indicate a dense distribution. In this study, ArcGIS 10.2 software was utilized to visualize the spatial distribution of point-like elements and reveal the spatial evolution process of spatial aggregation and dispersion of agritainment in China [39]. The formula used is

$$g(x)=\frac{1}{nh}\sum_{i=1}^{n}K\left(\frac{x-x_{i}}{h}\right)$$ (2)

where *x*_*i*_ is the coordinate of a point, *h* is the width, *k*(•) is a kernel function utilized to estimate the number and concentration of agritainment distribution points, and (*x* − *x*_*i*_) is the value of the distance from the estimated value point to the measured point.

#### 3.2.3. Disequilibrium index

The disequilibrium index is an indicator of the uniformity of the distribution of agritainment across the country. The disequilibrium index lies in the 0 − 1 range. The more aggregated the distribution of agritainment, the closer the value is to 1; and the more dispersed the distribution of agritainment, the closer the value is to 0 [39]. The formula used to calculate the index is

$$S=\frac{\sum_{i=1}^{n}Y_{i}-50\left(n+1\right)}{100n-50\left(n+1\right)}$$ (3)

where *n* is the total number of regions, *Y*_*i*_ is the cumulative percentage of the largest to smallest share of agritainment across the country, reflecting the balance of distribution of agritainment in the region. If *S* = 0, then agritainment is equally distributed in each region; but if *S* = 1, agritainment is fully concentrated in one region *S* ∈ (0,1).

#### 3.2.4. Exploratory spatial data analysis

Exploratory spatial data analysis is a combination of spatial data analysis techniques. It is a comprehensive analysis of the aggregation state of a research object in a geographic space. Exploratory spatial data analysis aims to reveal and analyze the interaction of a research object and an object in a geographic environment. In this study, Moran Getis-Ord $G_{i}^{*}$ and Moran’s I are selected to conduct spatial analysis of cold hotspots as well as spatial autocorrelation analysis of national agritainment [40]. The calculation formula is as follows:

$$I=\sum_{i=1}^{n}\sum_{i=1}^{n}W_{ij}\left(X_{i}-\bar{X}\right)\left(X_{j}-\bar{X}\right)/S_{j}^{2}\sum_{i=1}^{n}\sum_{i=1}^{n}W_{ij}$$ (4)

Because Moran’s I index indicates the global aggregation state of agritainment, Getis-Ordis $G_{i}^{*}$ is generally used in measurement analysis to identify local hot and cold spots of agritainment space distribution. The formula used to calculate Getis-Ordis $G_{i}^{*}$ is

$$I=\sum_{i=1}^{n}\sum W_{ij}\left(d\right)X_{j}/\sum_{i=1}^{n}X_{j}$$ (5)

By standardizing Getis-Ordis $G_{i}^{*}$,

$$Z\left(G_{i}^{*}\right)=\left[G_{i}^{*}-E\left(G_{i}^{*}\right)\right]/\sqrt{Var\left(G_{i}^{*}\right)}$$ (6)

is obtained, where X_i_ and X_j_ are the number of agritainment spots on the i and j geographic spaces, respectively. The spatial weight matrix is set to *W*_*ij*_, which is 1 when spatially adjacent and 0 when not. Moran’s I index is distributed between -1 and 1, and a value close to 1 indicates that there is similar attribute aggregation, a value close to -1 indicates a dissimilar attribute aggregation, and a value close to 0 indicates that there is no spatial autocorrelation [41].

$Var\left(G_{i}^{*}\right)$ is a variance value, $E\left(G_{i}^{*}\right)$ is an expectation value, and *W*_*ij*_(*d*) is the spatial weight matrix. If $Z\left(G_{i}^{*}\right)>0$ and passes the test, there is a high agglomeration in space and the agritainment in the area belongs to a hot spot. If $Z\left(G_{i}^{*}\right)$ is negative and passes the test, there is a low-value agglomeration in space and the agritainment in the area is in a cold spot [42].

#### 3.2.5. Geographical detector

Geographical detectors are a linear hypothesis method that can detect spatial differences and reveal drivers behind them using elegant shapes and clear physical meaning [43]. Geographical detectors are used to explore the intensity of different factors influencing the distribution of agritainment. The model is shown below

$$\begin{array}{l} q=1-\frac{\sum_{h=1}^{L}N_{h}\sigma_{h}^{2}}{N\sigma_{h}^{2}}=1-\frac{SSW}{SST}\\ SSW=\sum_{h=1}^{L}N_{h}\sigma_{h,}^{2}SST=N_{h}\sigma_{h}^{2} \end{array}$$ (7)

where h (h = 1, 2, …); L is variable Y or a factor of stratification; *N*_*h*_ is the number of cells in layer h; N is the number of cells in the full domain; $\sigma_{h}^{2}$ denotes the variance of layer h, *σ*^2^ denotes the variance of Y values in the full domain; SST denotes the total variance in the full domain; and SSW denotes the sum of variance within layers. q takes values of [0,1] and is proportional to the spatial differentiation of Y; when the stratification is generated by X, the larger the value of q, the stronger the explanatory power of X on Y and the weaker the opposite condition. When stratification is generated by X, the larger the value of q, the stronger the explanatory power of X on Y and the weaker the opposite condition [44].

## 4. Analysis of the spatial pattern of agritainment

### 4.1. Spatial differentiation features

#### 4.1.1 Characteristics of overall distribution

**Fig 2:**
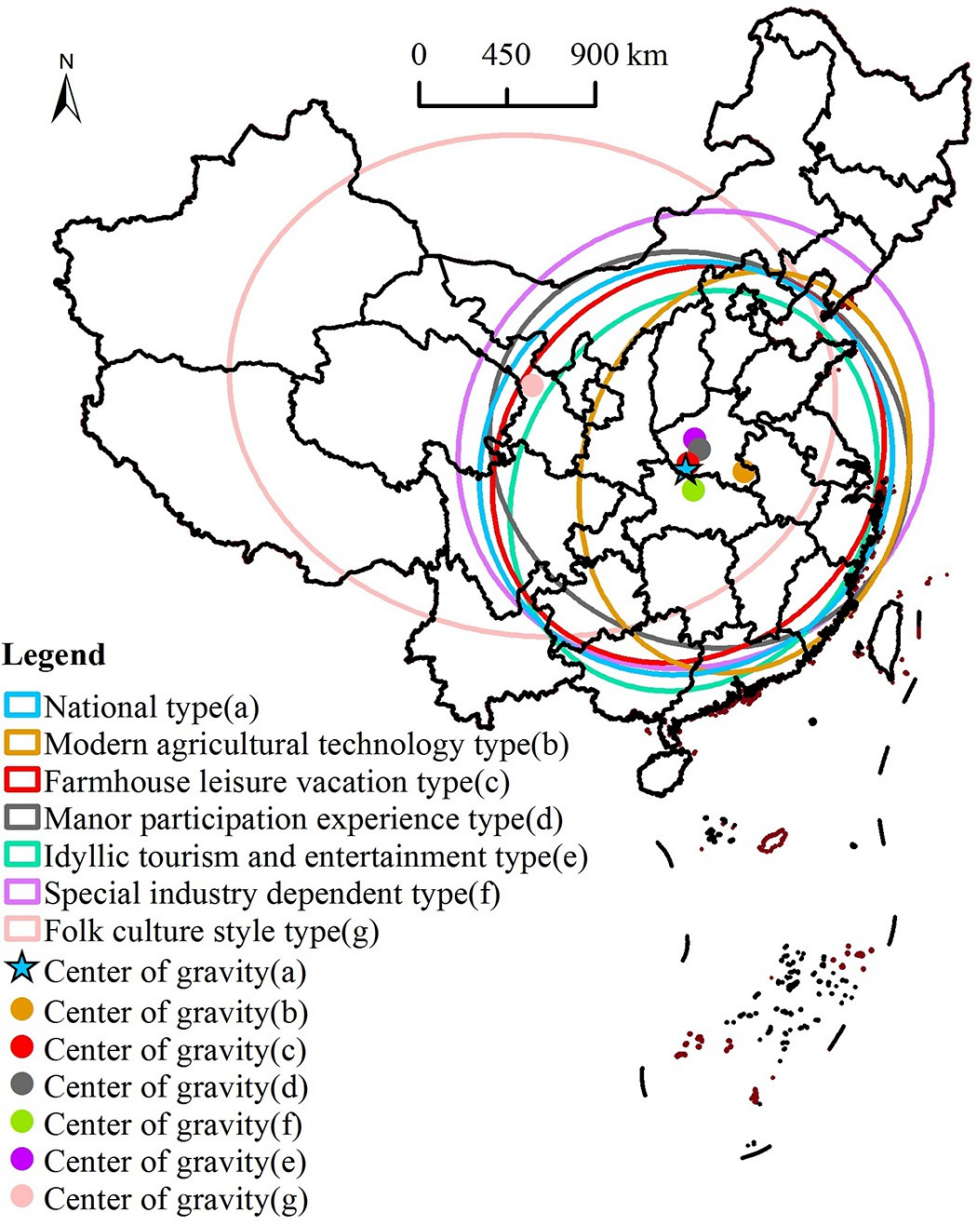
Standard deviation ellipse and center of gravity distribution of agritainment. Reprinted background map from the National Catalogue Service for Geographic Information (www.webmap.cn) under a CC BY license, with permission from the Ministry of Natural Resources of China, original copyright 2023.

Using the nearest neighbor index to illustrate the overall distribution type of national agritainment and its characteristics (Table 1), the calculation results show that the value of the nearest neighbor index of national agritainment is 0.236, which indicates a cohesive state in the geospatial distribution. The nearest neighbor indexes of various types of agritainment in China are concentrated between 0.337 and 0.489, and they all show a cohesive state. The smallest nearest-neighbor index is 0.337, which indicates the highest degree of agglomeration, while the largest nearest-neighbor index is 0.489, which indicates the lowest degree of agglomeration. Overall, the agglomeration degree of different types of agritainment has some variability, but they all present remarkable cohesive distribution characteristics. Moreover, the standard deviation ellipse is applied to explore the overall directional distribution characteristics of national agritainment (Fig 2). The ellipse of farm leisure vacation, manor participation experience, and rural sightseeing entertainment are roughly the same as that of all the country’s agritainment, and their spatial distribution, in general, shows a directional distribution pattern of northeast–southwest, and the central axis is roughly on the Beijing–Zhengzhou–Wuhan line. A large difference between the long and the short axes of the ellipse indicates a strong centripetal force and directionality of the spatial distribution. Among the six types of agritainment in China, the difference between the long and short axes of the ellipse of folk culture-type agritainment is the largest (the flatness of the ellipse is the largest), which means that its centripetal force and directional distribution is the strongest. Moreover, folk culture-type agritainment has a distribution along the northwest–southeast direction and a central axis that is roughly located in the Urumqi–Lanzhou–Wuhan line, revealing a regional distribution around the main gathering of ethnic minorities in China. The difference between the long and short axes of the standard deviation ellipse of special industry-dependent type is the least in size, and the directional distribution is not apparent.

#### 4.1.2. Characteristics of regional distribution

**Fig 3:**
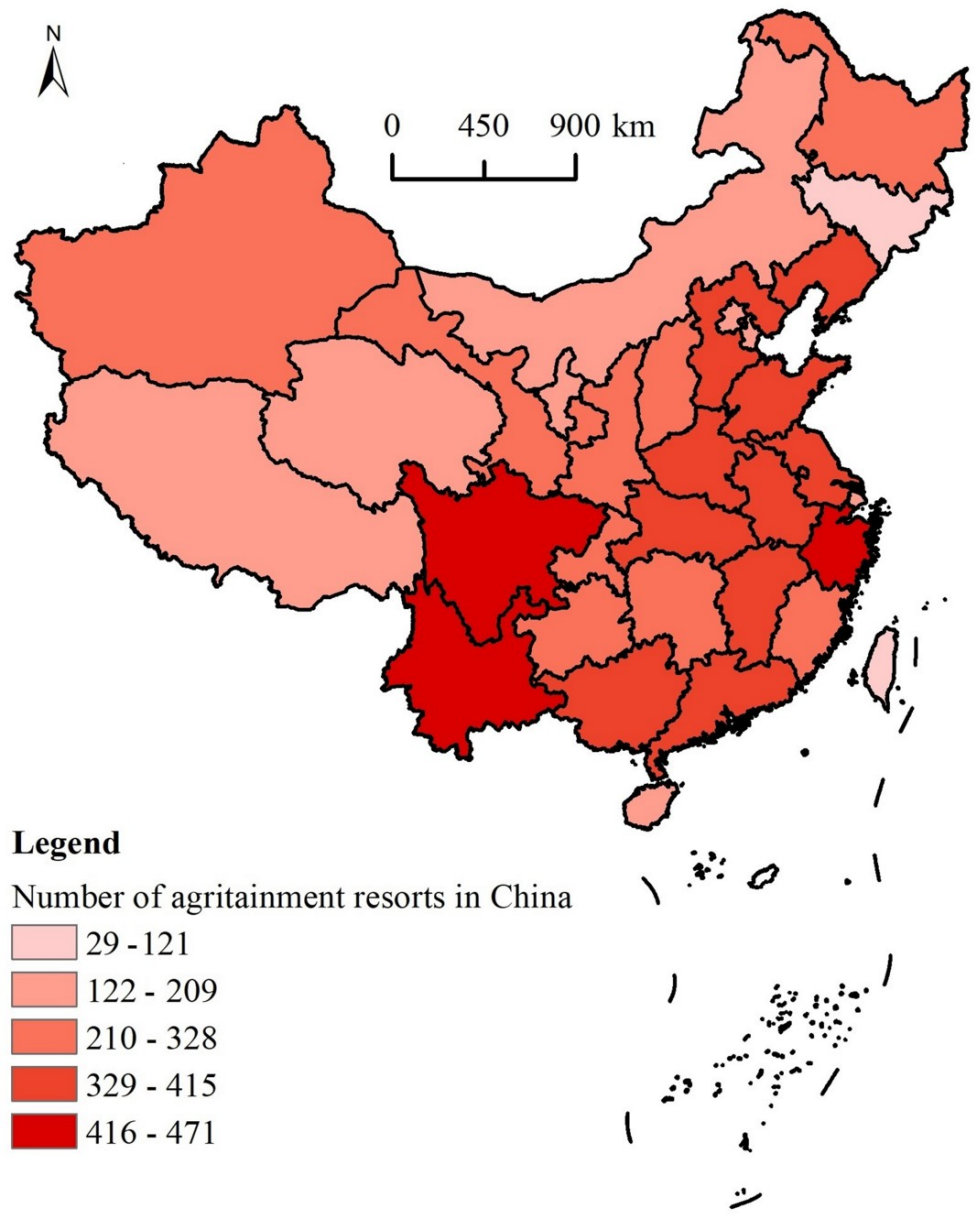
Differences in the distribution of provincial agritainment spots in China. Reprinted background map from the National Catalogue Service for Geographic Information (www.webmap.cn) under a CC BY license, with permission from the Ministry of Natural Resources of China, original copyright 2023.

**Fig 4:**
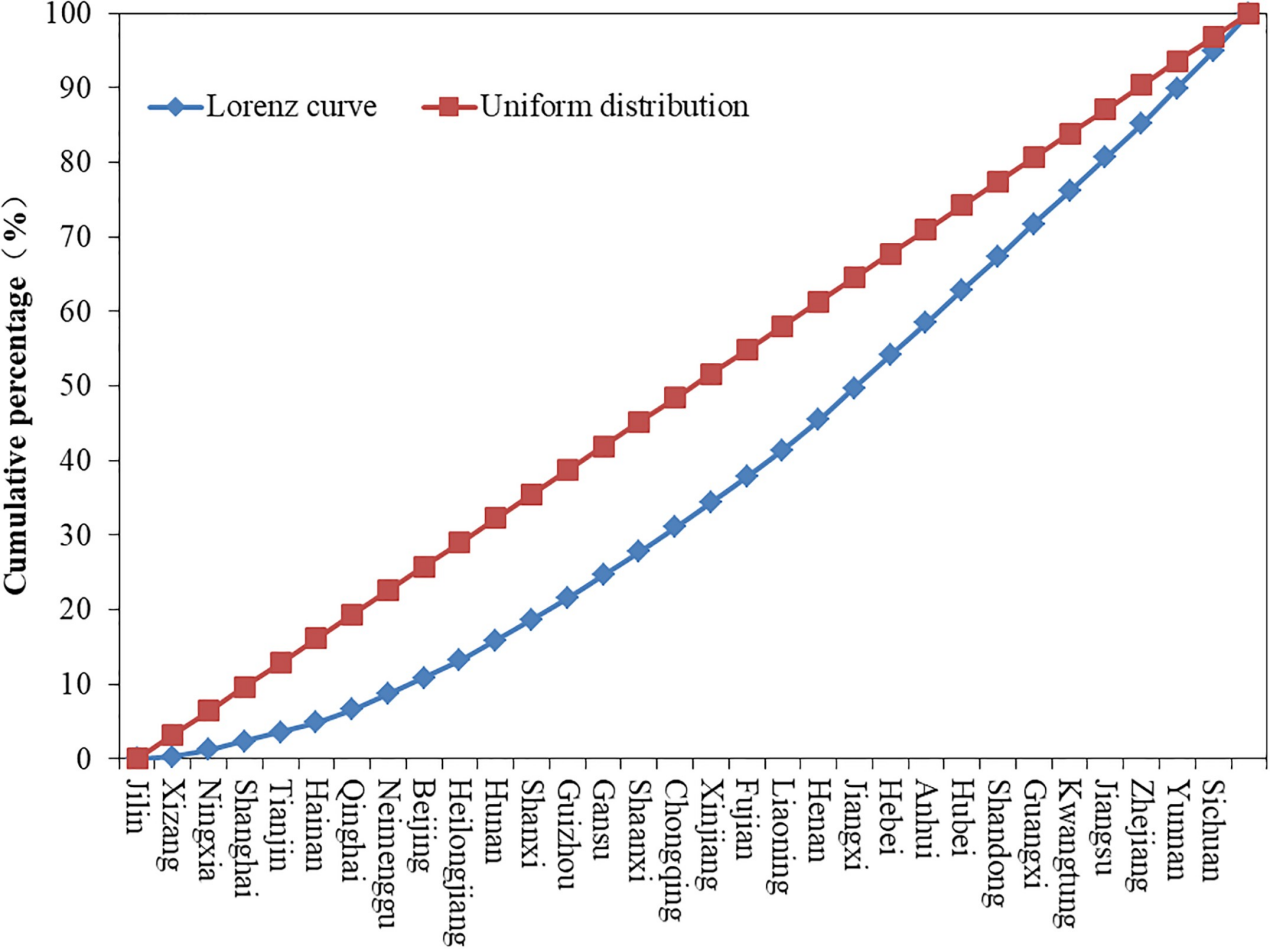
Spatial Lorentz curve of the distribution of agritainment.

In terms of the distribution of agritainment between 3 regions, the eastern region has the largest number of agritainment spaces: 3615, accounting for 39.3% of all the agritainment spaces in the country; the central region has 2599 farmhouses, accounting for 28.3% of the agritainment spaces in the country; and the western region has 2806 agritainment spaces, accounting for 30.5% of the agritainment spaces in the country. It is apparent that the distribution of agritainment in the three regions is heavier in the east and west and less heavy in the middle. The number of agritainment spaces in each province of China shows clear differences (Fig 3), and the disequilibrium index on the provincial scale is 0.024, which indicates that the unevenness of the distribution of agritainment on the provincial scale is more remarkable than it is in the eight regions. According to the Lorenz curve of the distribution of agritainment resorts in each province (Fig 4), it can be seen that there is a clear trend of a concave, indicating that the distribution of agritainment is particularly uneven in the following provinces and regions: Sichuan, Yunnan, Zhejiang, Guangdong, Jiangsu, and Guangxi. In these six provinces, the number of agritainment resorts is close to 30% of the national total. However, the number of agritainment resorts in Jilin, Tibet, and Ningxia provinces is low. Consequently, Chinese agritainment tends to be concentrated in provinces with a highly developed transportation economy and rich tourism resources.

### 4.2. Spatial density feature

**Fig 5:**
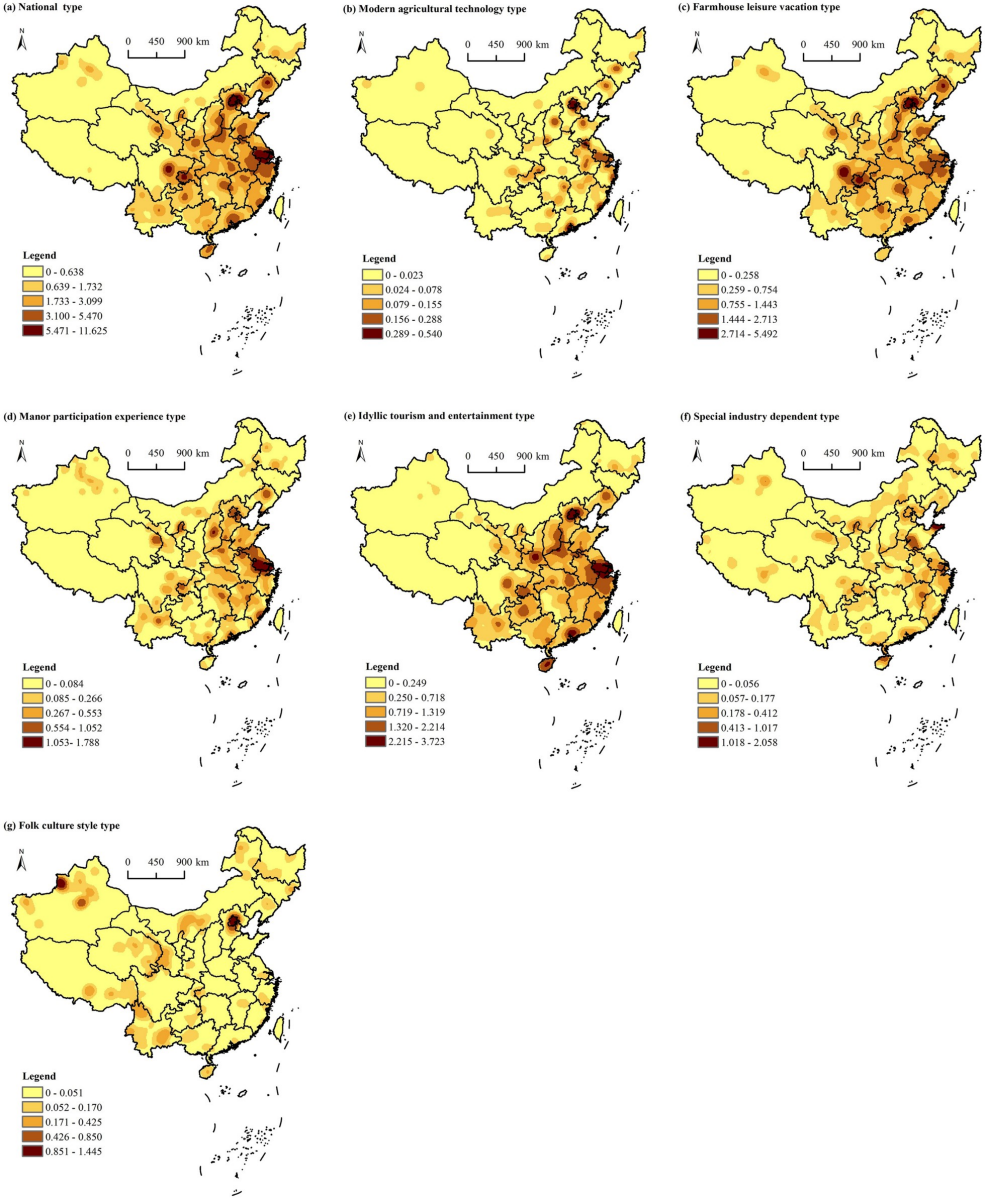
Kernel density distribution of agritainment in China. Reprinted background map from the National Catalogue Service for Geographic Information (www.webmap.cn) under a CC BY license, with permission from the Ministry of Natural Resources of China, original copyright 2023.

A kernel density analysis of agritainment using ArcGIS 10.2 software was utilized to generate a kernel density distribution map of national agritainment (Fig 5). In general, China’s agritainment has formed a high-density distribution with a core composed of Beijing–Tianjin–Hebei, Yangtze River Delta, and the Sichuan–Chongqing region; and a sub-high-density distribution area with the border of Shaanxi, Gansu, and Ningxia, the southern coast and the border of Hunan, Jiangxi and Hubei, showing clear spatial distribution characteristics of large agglomeration and small dispersion. High-density agritainment areas are located in eastern and southern of China, where the economy is well developed, the urbanization rate is high, the transportation between cities and towns is convenient, the urban population is large, and the pace of life is fast. Consequently, agritainment distributed in suburbs is loved by urban residents [45]. From the distribution density of different types of agritainment, the density of different types of agritainment is similar to the national distribution of agritainment in general, but there are also relative differences.

High-density areas of modern agricultural technology agritainment are mainly concentrated in the Yangtze River Delta, the Bohai Sea, and the southern coastal areas, while the sub-high-density areas are distributed in Hunan, Jiangxi, Hubei, Henan, Sichuan, Chongqing, Yunnan, Guizhou, Shaanxi, Gansu, and Ningxia. A small number of these high-density areas are distributed in the northwest. Most of these regions are economic and political centers or areas with high-tech industries, focusing on innovation and technology development. Farmhouse leisure vacation-type agritainment in Beijing–Tianjin–Hebei, the Yangtze River Delta, and the Sichuan–Chongqing region form a concentration center. These regions are economically developed areas of China, with high per capita income levels and sound infrastructure, which provide favorable conditions for the development of agritainment tourism. The high-density areas of manor participation experience agritainment are located in the Yangtze River Delta, Qinghai, Gansu, Ningxia, and Bohai Rim, which have a suitable climate for plant growth, high temperature, and rain in summer but mild and scant rain in the winter and sufficient light, a rich variety of vegetables and fruits, a well-developed planting industry, suitable for the development of agriculture and tourism combined with agritainment. Special industry-dependent agritainment is mainly concentrated in Shandong, the southern coastal region, Hainan, and forms sub-high density clusters in Inner Mongolia, Qinghai, Ningxia, et cetera. These regions are rich in fisheries, pastoralism, or special processing industries, and have thus facilitated the development of special agritainment tourism. Folk culture-style agritainment is mainly concentrated in the northwest, northeast, and southwest regions. These regions are the main concentrations of China’s ethnic minorities, with a variety of different characteristics of folk culture. Besides, the cultural atmosphere is strong. Therefore, folk customs facilitate the development of agritainment and can attract a large number of tourists.

### 4.3. Spatial correlation

**Fig 6:**
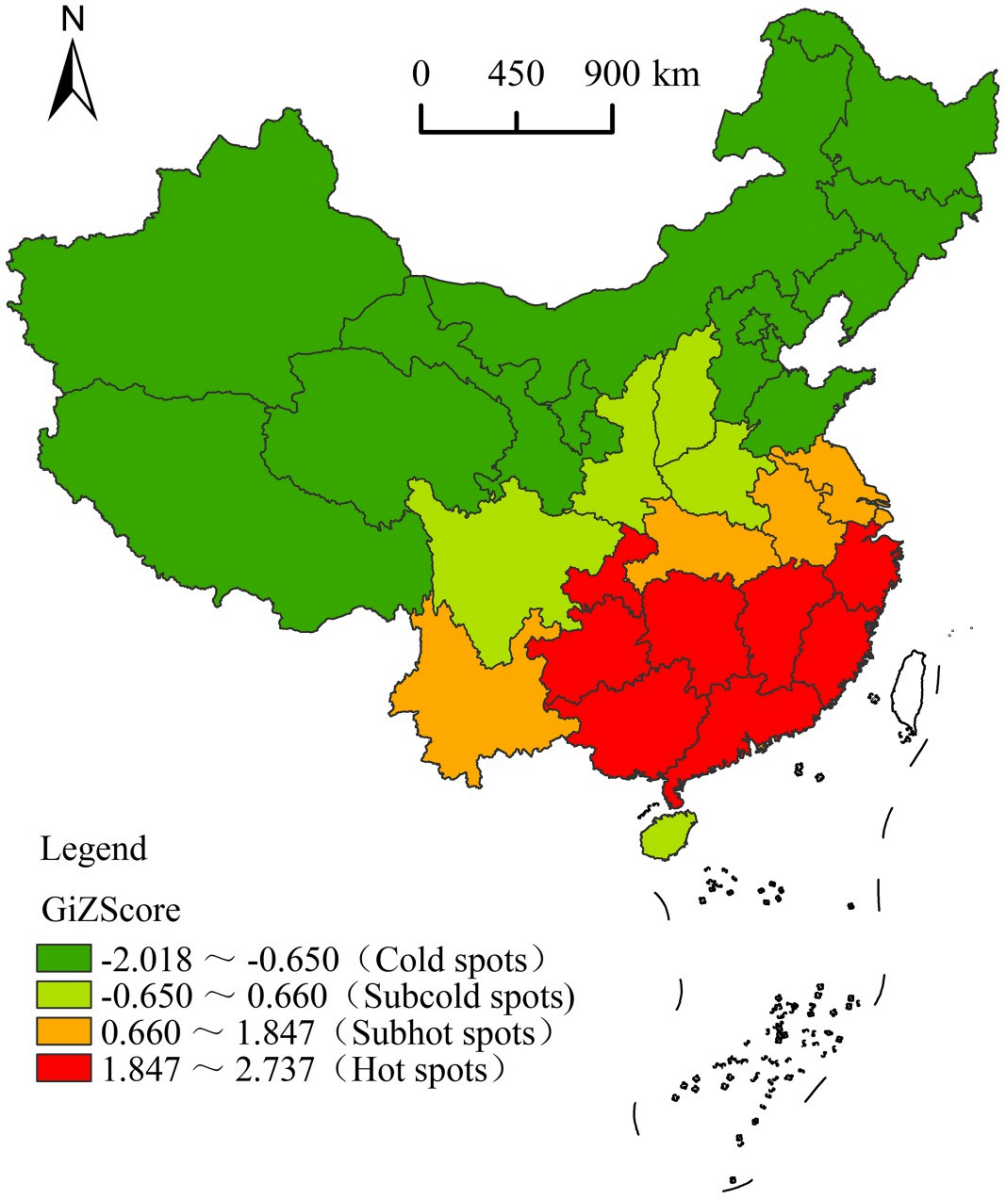
Regional distribution of agritainment cold spots and hot spots. Reprinted background map from the National Catalogue Service for Geographic Information (www.webmap.cn) under a CC BY license, with permission from the Ministry of Natural Resources of China, original copyright 2023.

**Fig 7:**
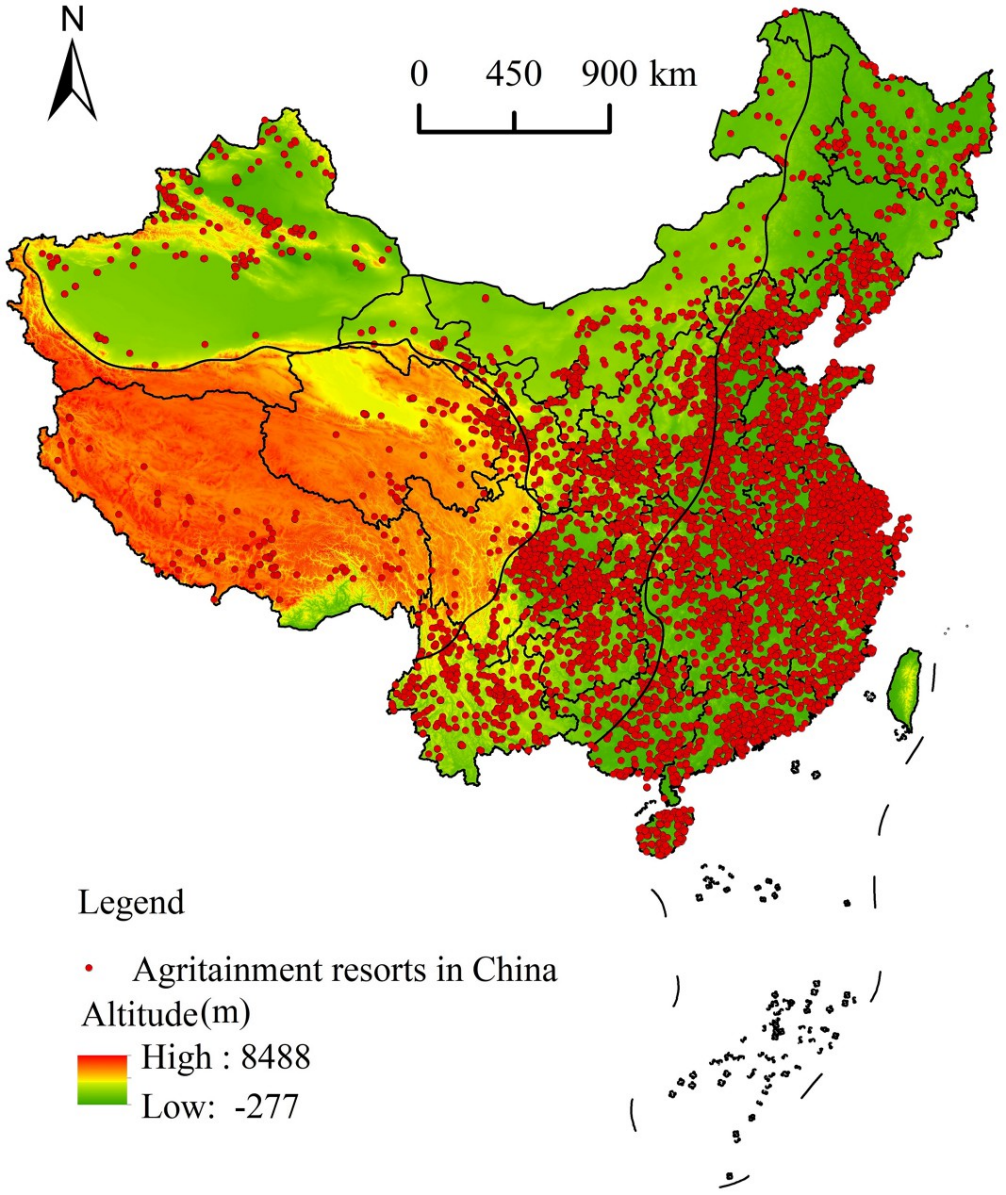
Agritainment in China and terrain overlay analysis. Reprinted background map from the National Catalogue Service for Geographic Information (www.webmap.cn) under a CC BY license, with permission from the Ministry of Natural Resources of China, original copyright 2023.

A spatial autocorrelation analysis of national agritainment was conducted using ArcGIS 10.2 software (Table 2), resulting in a Moran’s I index of 0.089. This value indicates that the degree of aggregation for agritainment in China was high, demonstrating a positive spatial autocorrelation. Subsequently, a spatial autocorrelation analysis was performed for each of the six types of agritainment found in China. Among these, Moran’s I for modern agricultural technology, manor participation experience, idyllic tourism, and entertainment and folk culture was greater than zero. The calculated z–value was positive, indicating a significant positive spatial correlation and suggesting that the development of these types of agritainment exhibited a spatial Matthew effect, with the spatial correlation of modern agricultural technology agritainment particularly showcasing this effect. On the other hand, Moran’s I for two types of agritainment (farmhouse leisure vacation and special industry—dependent agritainment) was less than zero, and their associated z-value was negative, indicating a significant negative spatial autocorrelation. The spatial difference for the special industry–dependent agritainment was the largest, showcasing a polarization effect in its spatial development. Additionally, a cold and hot spot identification analysis for agritainment and visual mapping (Fig 6) were conducted to explore the specific dynamics of spatial hot spot distribution of agritainment. Fig 7 illustrates that the distribution of cold and hot spots in the agritainment space exhibited a pattern characterized by "hot in the south and cold in the north." The hot spots and sub—hot spots in the south of China formed a continuous cluster distribution, and the cold spots and sub—cold spots mainly constituted a blocky cluster distribution in the northwest and northeast of China. There was a notable imbalance in the spatial development of agritainment in China. Therefore, optimization of the spatial layout is necessary to achieve balanced and sustainable development.

**Table 2:** Global Moran’s I index of agritainment in China.

	National Agritainment	Modern agricultural technology type	Farmhouse leisure vacation type	Manor participation experience type	Idyllic tourism and entertainment type	Special industry dependent type	Folk culture style type
Global Moran’s I Index	0.089	0.124	-0.046	0.021	0.142	-0.066	0.086
Expectation Index	-0.030	-0.033	-0.033	-0.033	-0.033	-0.033	-0.033
Variance	0.007	0.006	0.006	0.005	0.006	0.005	0.005
z-Value	1.460	2.018	-0.170	0.758	2.236	-0.493	1.650
p-Value	0.004	0.044	0.065	0.048	0.025	0.022	0.099

## 5. Factors influencing the spatial distribution of agritainment

Building upon research conducted by scholars such as Xiao Zhenquan and Wang Jing, this study delved into the factors influencing the spatial distribution of agritainment using four key concepts: economic, social, tourism industry, and natural. The examination encompassed the current state of the spatial distribution of agritainment nationwide [15]. The examined economic factors included regional gross production, per capita disposable income, per capita GDP, and per capita rural disposable income, while the social factors included comprehensive density of the highway network, comprehensive density of the railroad network, policy documents mentioning rural tourism, and the number of key villages engaging in rural tourism. The explored tourism industry factors included total tourism revenue, number of tourist receivers, number of scenic spots above level 4A, number of travel agencies, number of star-rated hotels, and the number of inns and lodges (as shown in Table 3). Analyzed natural factors included terrain, water bodies, climate, and precipitation. This study employed a geographic detector to assess the degree of influence of economic, social, and tourism industry factors on the spatial distribution of agritainment. Furthermore, the role of terrain, water, climate, and precipitation factors in the spatial distribution of agritainment was scrutinized using a geographic overlay analysis in GIS software to identify the factors affecting its spatial distribution.

**Table 3:** The construction of the index system of the humanistic category of agritainment.

Primary Influencing Factors	Secondary Influencing Factors
Economic factors	Gross regional production (X_1_)
	GDP per capita (X_2_)
	Disposable income per capita (X_3_)
	Rural disposable income per capita (X_4_)
Social factors	Comprehensive density of highways network (X_5_)
	Comprehensive density of railroad network (X_6_)
	Number of policy documents mentioning rural tourism (X_7_)
	Number of rural tourism key villages(X_8_)
Tourism Industry factors	Total tourism revenue (X_9_)
	Number of tourism receivers (X_10_)
	Number of scenic spots above 4A level (X_11_)
	Number of travel agencies (X_12_)
	Number of star-rated hotels (X_13_)
	Number of inns and lodges(X_14_)

### 5.1. Humanities factor analysis

#### 5.1.1. Factor detection analysis

The primary and secondary influencing factors affecting the spatial distribution of agritainment were analyzed using the geographic detector, with the q-value indicating the strength of influence of each indicator on the spatial distribution of agritainment. From Table 4, the intensity of the influence of the primary influencing factors on the distribution was as follows: tourism industry factors (0.69) > social factors (0.37) > economic factors (0.30). Thus, the tourism industry factors had the greatest influence, while the economic factors had the least influence on the distribution of agritainment. The q-values of secondary factors exhibited some variability, with the intensity of influence of four secondary factors (total tourism revenue, the number of tourism receivers, the number of scenic spots above level 4A, and the number of star-rated hotels) being above 0.5.

**Table 4:** Results of the detection of impact factors on the spatial distribution of agritainment in China.

Primary Influencing Factors	q-Value	Secondary Influencing Factors	q-Value
Economic factors	0.30	Gross regional production	0.40
		GDP per capita	0.14
		Disposable income per capita	0.12
		Rural disposable income per capita	0.11
Social factors	0.37	Comprehensive density of highways network	0.26
		Comprehensive density of railroad network	0.21
		Number of policy documents mentioning rural tourism	0.49
		Number of rural tourism key villages	0.49
Tourism Industry factors	0.69	Total tourism revenue	0.53
		Number of tourism receives	0.69
		Number of scenic spots above 4A level	0.51
		Number of travel agencies	0.38
		Number of star-rated hotels	0.64
		Number of inns and lodges	0.24

*(1) Economic factors*. The influence of economic factors on the spatial distribution of agritainment was relatively small. The explanatory power of the secondary influence factors (gross regional production, disposable income per capita, GDP per capita, and rural disposable income per capita) were 0.40, 0.12, 0.14, and 0.11, respectively. Among these four factors, gross regional production had the greatest influence on the spatial distribution of agritainment. This outcome indicates that areas with high regional GDP and high regional economic development levels had a higher number of agritainment attractions. Per capita disposable income, GDP per capita, and rural disposable income per capita reflect the degree of economic development of a region, influencing the spatial distribution of agritainment. When the regional economy is developed, the government and enterprises have additional funds to invest in the development and construction of agritainment attractions and aid residents in developing agritainment attractions. Therefore, a region’s level of economic development significantly influences the spatial distribution and development of agritainment [46]. In some economically underdeveloped areas, there are few agritainment attractions due to inadequate investments in construction; consequently, the measured level of the development level of agritainment is low.
*(2) Social factors*. Social factors exert a relatively significant influence on the development and distribution of agritainment. Among the secondary influencing factors, the explanatory power of the highway network and the railway network comprehensive density was 0.26 and 0.21, respectively. These values suggest that these two factors have some influence on the spatial distribution of agritainment. Robust transport conditions are crucial for tourism development as they serve as links between tourist sources and destinations. Agritainment resorts situated in areas with underdeveloped transport infrastructure witness restricted growth. Consequently, these areas need to improve their transport infrastructure, enhance road construction, improve accessibility, and guarantee road safety to foster a flourishing agritainment tourism industry. The explanatory power of the number of policy documents mentioning rural tourism and the number of key villages engaging in rural tourism in each province as secondary influencing factors are 0.49. A substantial number of both is indicative of a region’s importance and support for tourism development in contributing to rural revitalization. As the primary factor influencing rural tourism development, agritainment tourism can assist in addressing the “agriculture, rural areas, farmers issues,” effectively optimizing the rural industrial structure, promoting the overall development of the rural economy, life, and culture, and facilitating rural urbanization and industrialization. All provinces in China have responded positively to the policy, instituting pertinent policies to revitalize the development of rural agritainment. They have identified key villages for rural tourism promotion, safeguarded key development objectives, invested substantial material and financial resources to enhance the improvement of tourism services and facilities, facilitated tourism development planning, and encouraged individuals in the regions to actively participate in the development of the agritainment industry [47].
*(3) Tourism industry*. Tourism industry factors have the most significant influence on the spatial distribution of agritainment. Because of their secondary influencing factors, the explanatory powers of the total tourism revenue, the number of tourist receptions, the number of scenic spots above level 4A, and the number of star-rated hotels are 0.53, 0.69, 0.51, and 0.64, respectively. These values indicate that these four factors exert considerable influence on the spatial distribution of agritainment. Tourism income serves as a valuable indicator of the degree of tourism development and the economic benefits associated with tourism in a given region. High overall incomes facilitate the development of tourism. The more developed the local tourism industry, the stronger the support for agritainment, as reflected in increased tourist traffic.

The tourism receivers index reflects the flow of tourists in an area, correlating with the demand for tourism products. Given that agritainment depends on a large number of tourists to remain profitable, the availability of tourists significantly influences its distribution. The number of 4A-level scenic spots in a region is indicative of the quality of tourism resources, with quality playing a considerable role in shaping the distribution of agritainment. Consequently, a surplus of high-quality tourism resources and scenic spots enhances agritainment development. As intermediaries linking tourist sources and destinations, travel agencies play a pivotal role in influencing the growth and development of agritainment. The number of travel agencies focusing on a given region is indicative of the local tourism development level. Therefore, travel agencies can channel additional tourists from diverse sources into a specific region’s agritainment industry, thereby impacting its growth [48]. Another crucial factor in the development of agritainment in an area is the presence of inns and lodges, which are major sub-products of agritainment. Investors determine the number of inns and lodges around a scenic spot, thus exerting a significant impact on the regional distribution of agritainment. As a region advances its tourism industry, the number of inns and lodges associated with agritainment increases.

#### 5.1.2. Interaction detection analysis

Interaction detection analysis was conducted using a geographic detector to examine the degree of influence of two-factor interaction on the spatial distribution of agritainment nationwide. From the results (Table 5), single—factor interaction in this study produced a two-factor enhanced and a non-linear enhanced type, indicating that the combination of two factors had more explanatory power than the single-factor effect in the spatial distribution of agritainment. The result suggests that the development of agritainment is influenced by the interaction of multiple factors [19]. Among the examined single factors, factors on policy requirements for rural tourism (X_7_), the number of tourist receivers (X_10_), the number of scenic spots above level 4A (X_11_), and the number of star-rated hotels (X_13_) interacted with other factors to yield q-values above 0.7, indicating their substantial contribution to the overall impact of the combination of factors on agritainment. Therefore, compared to the others, the single factors listed above had a greater two-factor interaction impact. Among the two-factor interactions of each group, the interaction between the number of scenic spots with a rating above level 4A and the number of star-rated hotels (X_11_∩X_13_) had the strongest interaction effect with a combined q-value of 0.92. The result indicates that the combination of scenic spots with a rating above level 4A and star-rated hotels have a significant influence on the development of agritainment in a region. The strength of the influence is also indicated by the fact that single-factor q-values of the number of scenic spots rated above level 4A and the number of star-rated hotels were as high as 0.51 and 0.64, respectively. The result also indicates that the q-values of the two factors, which had high single-factor q-values, remained high after the interaction.

**Table 5:** Results of the interaction detection of spatial differentiation of agritainment in China.

	X_1_	X_2_	X_3_	X_4_	X_5_	X_6_	X_7_	X_8_	X_9_	X_10_	X_11_	X_12_	X_13_	X_14_
X_1_	0.40													
X_2_	0.70	0.14												
X_3_	0.63	**0.20**	0.12											
X_4_	0.72	0.41	**0.21**	0.11										
X_5_	0.67	**0.37**	0.57	0.44	0.26									
X_6_	0.62	0.39	0.44	0.35	0.49	0.21								
X_7_	**0.65**	**0.58**	**0.60**	0.61	**0.60**	**0.57**	0.49							
X_8_	**0.74**	0.66	0.69	0.65	**0.65**	0.77	**0.83**	0.49						
X_9_	**0.67**	0.76	0.69	0.78	0.80	0.79	**0.84**	**0.64**	0.53					
X_10_	**0.75**	**0.77**	0.82	0.82	**0.85**	**0.85**	**0.81**	**0.81**	**0.80**	0.69				
X_11_	**0.68**	0.74	0.83	**0.59**	**0.63**	0.88	**0.75**	**0.86**	**0.87**	**0.86**	0.51			
X_12_	**0.68**	0.60	0.57	0.56	0.81	0.67	**0.76**	**0.61**	**0.67**	**0.78**	**0.82**	0.38		
X_13_	**0.74**	0.84	0.82	0.77	**0.84**	**0.81**	**0.79**	**0.74**	**0.73**	**0.83**	**0.92**	**0.71**	0.64	
X_14_	0.65	0.55	0.58	0.50	0.64	0.64	0.80	**0.56**	**0.67**	**0.77**	0.77	**0.43**	**0.70**	0.24

Note: Underlined data are one-factor effects; bold data are two-factor enhanced q(X_1_ ∩ X_2_) > max(q(X_1_), q(X_2_)) for interactions; the rest are non-linear enhanced q(X_1_ ∩ X_2_) > q(X_1_) + q(X_2)_ for interactions.

### 5.2. Analysis of natural factors

#### 5.2.1. Terrain

China has numerous types of terrain, including plains, hills, plateaus, deserts, basins, and mountains. To identify the correlation between agritainment and terrain in China, this study used ArcGIS 10.2 software to overlay the terrain elevation map of China on a map of the spatial distribution of agritainment resorts in China (Fig 7). This study found 7143 agritainment resorts (accounting for 77.6% of all agritainment resorts) in the three-step area below 1,000 m above sea-level, 1465 agritainment resorts (15.9%) in the three-step area between 1000 and 2000 m above sea-level, 408 agritainment resorts (4.4%) between 2000 and 4000 m above sea-level, and only 63 agritainment resorts (0.7%) above 4,000 m above sea-level. The spatial distribution of agritainment resorts in the area was also investigated. In addition, using SPSS, a correlation analysis was performed on the number of agritainment resorts and their attendant elevation values. The analysis produced a correlation coefficient of -0.978, which is a significant negative correlation, indicating that the number of agritainment resorts gradually decreased with the elevation. Therefore, the distribution of agritainment resorts is strongly correlated with elevation [49]. The result suggests that road construction in high altitudes and rugged terrain should be accelerated. Improving road transport is the first step towards increased tourist traffic in a destination, and it is a major influencing factor in the development and growth of agritainment.

#### 5.2.2. Water body systems

**Fig 8:**
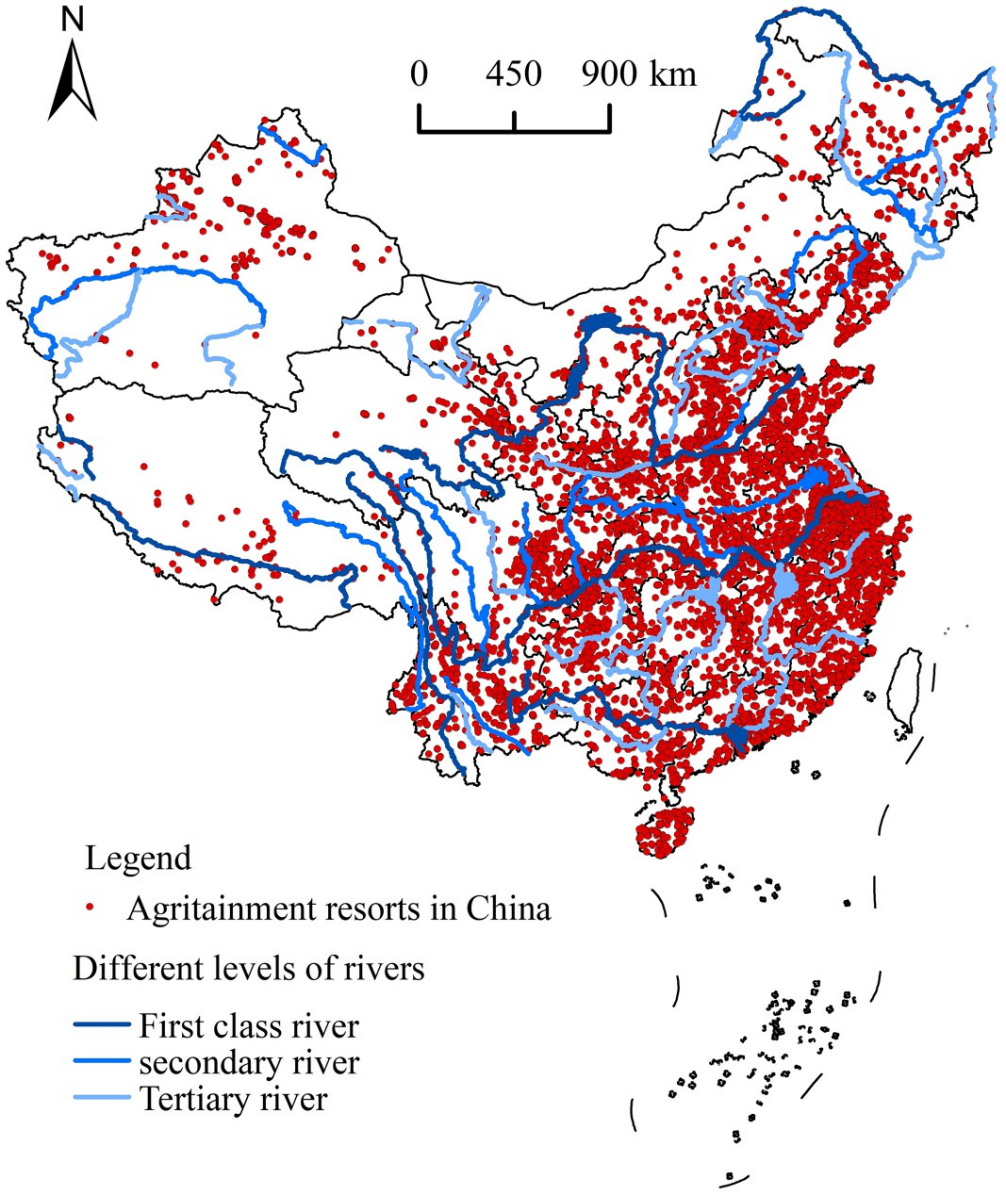
Agritainment in China and major river overlay analysis. Reprinted background map from the National Catalogue Service for Geographic Information (www.webmap.cn) under a CC BY license, with permission from the Ministry of Natural Resources of China, original copyright 2023.

Water bodies in the region constitute a major factor affecting the geographical distribution of agritainment resorts. Most rural tourism destinations have established ecological balance with natural resources and are strategically located near water bodies. Consequently, they are accessible by water, creating a unique form of rural tourism that directly impacts the spatial distribution of agritainment resorts. In Fig 8, the national spatial distribution map of agritainment resorts is overlaid with the primary, secondary, and tertiary rivers in China. ArcGIS 10.2 software was utilized to analyze the buffer zones of rivers with a radius of 5–15 km and to count the number of agritainment resorts in different river buffer zones. The results revealed that there were 994 agritainment resorts (accounting for 10.8% of all agritainment resorts) within the 5 km buffer zone of the river, 1630 agritainment resorts (17.7%) within the 10 km buffer zone of the river, and 2449 agritainment resorts (26.6%) within the 15 km buffer zone of the river. Additionally, the correlation between the distance of the buffer zone and the number of agritainment resorts within the buffer zone was analyzed, yielding a correlation coefficient of 0.997, indicating a significant correlation. Therefore, the evidence suggests that the number of agritainment resorts in the buffer zone increases with the size of the buffer zone and the resorts are predominantly distributed around the river course. Having a well-established water body is a crucial determining factor for the formation of excellent tourism resources, making water bodies indispensable to the development of quality agritainment tourism services. In general, water bodies are vital resources that enhance the development of agritainment services. Therefore, the water system is a crucial factor affecting the spatial distribution of agritainment resorts.

#### 5.2.3. Climate

**Fig 9:**
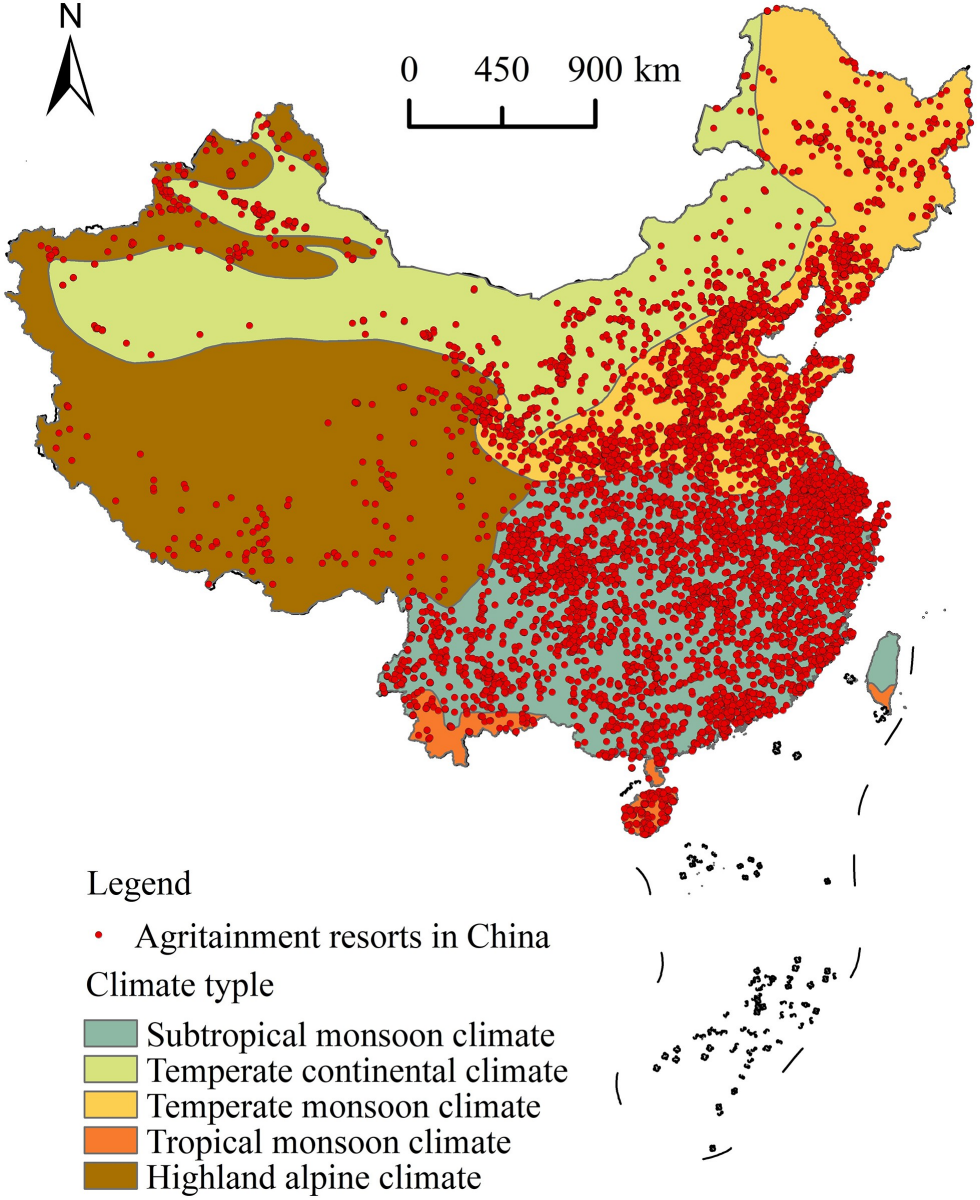
Agritainment in China and climate overlay analysis. Reprinted background map from the National Catalogue Service for Geographic Information (www.webmap.cn) under a CC BY license, with permission from the Ministry of Natural Resources of China, original copyright 2023.

Climate is an important influencing factor in the creation of tourism resources because it strongly determines whether tourists choose a destination. Climate determines the temperature and precipitation patterns experienced in a region, which influence the development of tourism resources. ArcGIS 10.2 software was used to overlay the spatial distribution map of national agritainment resorts on the climatic zone map of China (Fig 9). Statistical analysis revealed that there were 7862 agritainment resorts (accounting for 85.5% of all agritainment resorts) in the subtropical monsoon and temperate monsoon climate regions, making the number of agritainment resorts distributed in the subtropical monsoon climate region the largest: 5021 (54.6%), while the temperate monsoon climate region had 2841 agritainment resorts (30.9%). There were 665 agritainment resorts (7.2%) in the temperate continental climate zone and some (4.7%) in the highland mountain climate zone. The number of agritainment resorts in tropical monsoon climate zone was at least 227 (2.5%). The monsoon climate has four distinct seasons that are associated with four periods that have relatively different types of natural scenery: Summer is hot and rainy, and winter is cool [50]. When climatic conditions are inviting, tourists have a strong incentive to visit destinations. Therefore, the quality of climatic conditions is an important factor influencing the development of agritainment tourism and affects the distribution of agritainment and the decision-making of tourists regarding places they visit.

#### 5.2.4. Precipitation

**Fig 10:**
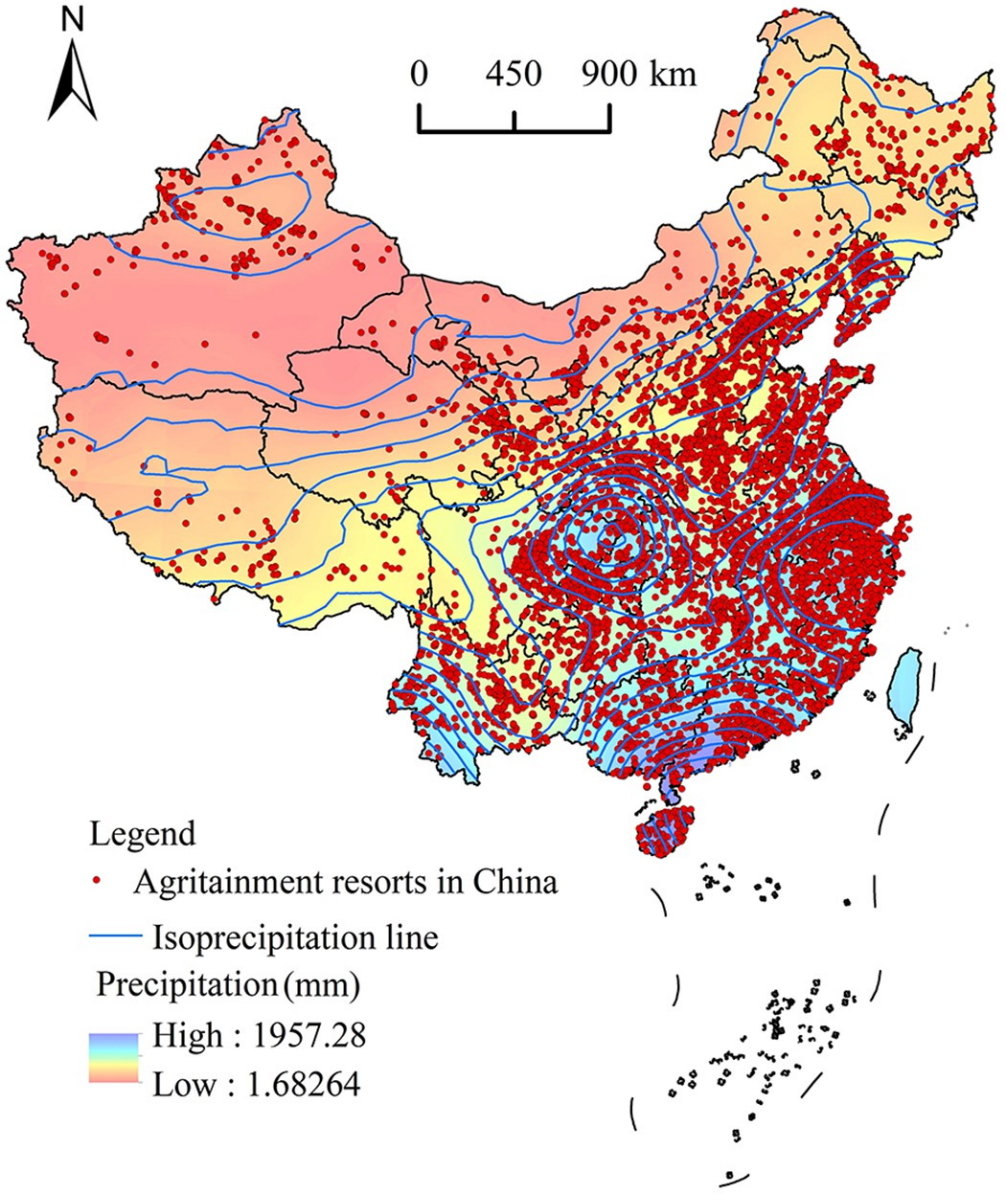
Agritainment in China and rainfall precipitation overlay analysis. Reprinted background map from the National Catalogue Service for Geographic Information (www.webmap.cn) under a CC BY license, with permission from the Ministry of Natural Resources of China, original copyright 2023.

Precipitation levels have a strong correlation with vegetation type and the ecological environment of an area. To investigate the influence of precipitation on the spatial distribution of agritainment resorts, a spatial distribution map of national agritainment resorts was overlaid on a national annual precipitation map using ArcGIS 10.2 software (Fig 10). Based on the precipitation received, the country was divided into four zones: arid, semi-arid, semi-humid, and humid. The number of agritainment resorts within these four areas was counted. Among them, the humid zone had the highest number of agritainment resorts at 4217 (accounting for 45.8% of all agritainment resorts), while there were 3237 resorts in the semi-humid zone (35.2%), 1040 resorts in the semi-arid zone (11.3%), and 706 resorts in the arid zone (7.7%). This result indicates that agritainment resorts are mainly established in humid and semi-humid areas that have high precipitation. To further investigate the relationship between precipitation and the distribution of agritainment, a correlation analysis between the variables was performed, obtaining a correlation coefficient of 0.968. The high correlation coefficient suggests that annual precipitation has a significant influence on the spatial distribution of agritainment resorts. The amount of precipitation an area receives determines the diversity of its natural vegetation, which affects the distribution of agritainment resorts [51].

## 6. Discussion

### 6.1. Research findings compared to other studies

The development of the rural tourism industry can employ residents, helping farmers in disadvantaged areas fight poverty by earning adequate incomes. Therefore, improving rural tourism has become an important strategy in China’s efforts of rural revitalization, receiving extensive attention and strong support from governments at all levels [52]. Research on rural tourism has become a topic of interest in recent years in academic research. Recent research in China and other parts of the world has examined rural tourism from a spatial perspective, with a primary focus on picking gardens, leisure agriculture demonstration sites, leisure villages, key rural tourism villages, and rural homestays [53–57]. However, there is a need for a systematic exploration of the spatial distribution and factors affecting agritainment, particularly through a large-scale systematic study of the country.

To investigate the planning and factors affecting agritainment and its spatial distribution, this study selected agritainment in 31 Chinese provinces as a research object. The study reveals that the spatial distribution of agritainment resorts is relatively apparent, with high-density areas such as the Yangtze River Delta, Beijing–Tianjin–Hebei, and Sichuan–Chongqing regions serving the core of agritainment in the country. Therefore, the distribution of agritainment resorts in nationwide is relatively apparent, while the distribution of agritainment resorts in the inter-region is relatively imbalanced, aligning with findings from previous studies [53]. This study systematically investigates the spatial distribution of agritainment: its results can be used to optimize the spatial layout of rural tourism and enhance its sustainable development.

The spatial distribution of agritainment is affected by many factors. This paper analyzes the factors that influence the spatial distribution of agritainment from two dimensions: human and natural factors. Using an index system of humanistic factors of economy, society, and tourism industry, a geographical detector was used to explore the intensity of influence of different indicators. To enhance the reliability of the research results, an interactive analysis of each factor was performed to explore the interaction between factors. It was found that the interaction between the number of level 4A scenic spots and the number of star-rated hotels had the strongest influence on the spatial distribution of agritainment resorts, indicating that the development of agritainment is significantly affected by a region’s scenic spots and accommodation facilities. Generally, the number of scenic spots and accommodation facilities in a region is indicative of its level of tourism development, with a high level of regional tourism development suggesting a strong driving role of regional agritainment development. For example, Sandt et al., found that scenic spots have a strong driving effect on the development of rural tourism in the surroundings, and they can create a beneficial synergistic development effect with rural tourist destinations [58].

The effects of natural factors on the spatial distribution of agritainment were analyzed using geographical superposition and buffer analysis on four factors: terrain, precipitation, water systems, and climate. It was found that most agritainment resorts were distributed in plain and hilly areas with high humidity, an altitude of less than 1000 m, and annual precipitation of more than 800 mm. Most of the agritainment resorts are located close to rivers in subtropical monsoon climate areas with suitable climates.

This study suggests that hospitable regional geographical conditions positively influence the development of agritainment. Generally, the suitability of a geographical location impacts regional social and economic development and population distribution, with a relatively good geographical location supporting farming relatively easily. This finding is consistent with that of Shaken et al. [59–61]. This systematic study of the factors affecting agritainment provides an evidence-based reference that can aid its sustainable development.

### 6.2. Research limitations

This study has the following limitations: (1) Due to a lack of panel data on agritainment, no investigation has been done on the spatiotemporal evolution of agritainment. In recent years, China’s rural tourism industry has developed rapidly, making it an important product. An accurate characterization of the spatiotemporal development of rural tourism would function as a useful reference for enhancing the sustainable development of rural tourism in China. Future research could focus on collecting data on agritainment through the years to systematically investigate the spatiotemporal evolution of agritainment. (2) Further research on the factors influencing agritainment is needed. This paper offers a relatively comprehensive systematic exploration of the spatial factors influencing agritainment from two dimensions: humanity and nature. The spatial distribution of agritainment is also affected by other factors, including tourist preferences, operator management level, residents’ participation, and farmers’ entrepreneurial willingness and professional skills, which are likely to have a significant impact on the spatial distribution of agritainment. In the future, a multi-dimensional index system of influencing factors should be built. In this study, the factors influencing the spatial distribution of agritainment were systematically explored qualitatively and quantitatively. (3) This study has not investigated the negative effects of agritainment. Because it is a form of rural tourism, the development of agritainment is likely to be associated with specific cultural conflicts, environmental pollution, and land-grabbing issues. These negative effects are also likely to influence the spatial distribution of agritainment. These research limitations can be addressed in future research, improving the quality of available information on agritainment.

### 6.3. Practical implications

This study clarifies the spatial distribution characteristics of Chinese agritainment from the perspective of geographic space, and systematically explores the influencing factors of its spatial distribution. The research results have certain guiding significance for the high-quality development of agritainment.

First, optimize spatial layout. At present, agritainment shows a spatial distribution of "more east and west, less central" in the country, the future should increase the distribution of agritainment in the central region, and gradually realize the balanced development of agritainment, so as to better meet the needs of consumers in different regions of agritainment tourism products.

Second, the construction of agritainment tourism cluster. In the future, it is necessary to strengthen the inter-regional linkage development, promote the coordinated development of agritainment in Beijing-Tianjin-Hebei, Yangtze River Delta and Sichuan-Chongqing regions, and form a agritainment tourism cluster with world influence.

Third, we will foster agritainment demonstration cities. The spatial distribution of agritainment has a certain direction and is distributed around key cities. In the future, efforts should be made to build a number of national agritainment tourism nodes and demonstration cities such as Beijing, Zhengzhou and Wuhan to achieve the demonstration and leading effect of agritainment development.

Fourth, development with local characteristics. From the analysis of influencing factors of the spatial distribution of agritainment, it can be seen that tourism industry factors have a great impact on the spatial distribution of agritainment. In the future, all regions should continuously improve the development quality of their cultural tourism industry and comprehensively help further improve the development level of regional agritainment. Secondly, the study shows that the interaction between the number of 4A-level scenic spots and the number of star-rated hotels has a great impact on the spatial distribution of agritainment. In the future, the number of high-grade tourist attractions and star-rated hotels in the region should be continuously increased, so as to create conditions for the development of agritainment in the region and realize the coordinated development of each other. Finally, local agritainment should be based on local characteristics of traditional culture, intangible cultural heritage, food culture and other cultural tourism resources endowment and unique natural geographical conditions, according to local conditions to promote the coordinated development of agritainment and rural homestays, rural scenic spots, rural resorts, rural scenic paths and rural tourism destinations, so as to achieve differentiation, characteristics and sustainable development of rural tourism [62–64].

This study is a further extension of Cui and Flanigan scholars’ research [65, 66]. First, it divides the types of agritainment for the first time, and studies the spatial distribution characteristics of different types on this basis. Second, the paper constructs the factors influencing the spatial distribution of agritainment from two dimensions of nature and humanity, among which the humanistic factors are all constructed with composite indicators, which is innovative. Third, the integrated use of a variety of spatial analysis methods. In this paper, various GIS spatial analysis techniques such as nearest neighbor index, standard elliptic equation, kernel density, disequilibrium index and exploratory spatial data analysis are comprehensively used to systematically explore the spatial distribution characteristics of Chinese agritainment. It is innovative to systematically identify the main factors of the spatial distribution of Chinese agritainment by comprehensive methods such as reason detector, spatial superposition and buffer analysis. Agritainment can help bring about rural revitalization in China if it is used effectively to promote social and economic development in disadvantaged areas [49]. Agritainment is an important carrier for the implementation of rural revitalization strategy in China, and an effective way to promote social and economic development in poor areas. In the future, agritainment should give more play to their powerful function of helping rural revitalization, improve villagers’ ability to obtain tourism income, and create more and better development opportunities for them so that tourism development and beautiful countryside construction can go hand in hand. For example, this study only selects gold medal agritainment for analysis, but does not classify the agritainment into classes, and further investigate the influence of other factors on the distribution of agritainment, these research directions will be carried out in the next study.

## 7. Conclusion

1. Overall Distribution—Identifiable patterns were observed in the spatial distribution of all types of agritainment in China. The highest degree of spatial agglomeration was found in categories of farmhouse tourist establishments, and the lowest degree of spatial agglomeration was found in the characteristics of industry dependence. An analysis of all agritainment establishments in the country revealed a "northeast–southwest" direction distribution trend, with the central axis generally located along the "Beijing–Zhengzhou–Wuhan" line. The direction of the distribution of different types of agritainment establishments features identifiable variations.
2. Regional Distribution—Variations were observed in the distribution of agritainment establishments at different spatial scales. Among the three major zones, a spatial distribution pattern characterized by "more in the east and west and less in the center" was observed. Analysis of the provincial distribution of agritainment resorts indicated they are primarily concentrated in six provinces, namely Sichuan, Yunnan, Zhejiang, Guangdong, Jiangsu, and Guangxi, with relatively smaller numbers of resorts found in three provinces, namely Jilin, Tibet, and Ningxia.
3. Spatial Density Distribution—The country has several high-density areas, including the core areas, the Beijing–Tianjin–Hebei region, Yangtze River Delta, and Sichuan–Chongqing regions. Other regions can be categorized as sub-high-density distribution areas, including the Shaanxi–Gansu–Ningxia border region, the southern coastal region, and the Xiangan–Jiang–Hubei border region, which has a population distribution characterized by the pattern "large agglomeration, small dispersion." The distribution and density of different types of agritainment resorts are relatively similar to the distribution and density of the population of the country, but there are some differences.
4. Spatial Correlation–The national agritainment sector exhibits significant and positive spatial autocorrelation, with the Matthew effect being relatively pronounced in the region. Agritainment cold and hot spots display a distribution characterized by the pattern "hot in the south and cold in the north." The hot spots and sub-hot spots in the south of China demonstrate a continuous cluster distribution pattern, whereas the cold spots and sub-cold spots mainly exhibit a blocky cluster distribution pattern in the northwest and northeast of China. Additionally, while leisure vacationing and the industry-dependent types of agritainment resorts show a negative spatial autocorrelation, other types of agritainment resorts have a positive spatial autocorrelation.
5. Influencing Factors—The spatial distribution of agritainment resorts in China is jointly affected by human factors such as society, the economy, and the tourism industry as well as natural factors such as terrain, water body systems, and climate. The extent of influence of the first-level humanistic factors on the spatial distribution of agritainment resorts is as follows: tourism industry factors (0.69) > social factors (0.37) > economic factors (0.30). Among the second-level index factors, the total tourism income, the number of tourist receivers, the number of 4A-level tourist attractions, and the number of star-rated hotels have the greatest effect on the spatial distribution of agritainment. The results of factor interaction analysis indicate that the interaction value increased between different combinations of two factors and the spatial distribution of agritainment resorts. The dual-factor number of level 4A scenic spots has the strongest interaction effect on the number of star-rated hotels (X_11_∩X_13_). An analysis of natural factors indicated that the agritainment resorts are primarily distributed in humid plains and hilly areas below 1000 m above sea level, in areas where the annual precipitation is more than 800 mm. Consequently, the resorts are mostly distributed in areas close to a river in subtropical monsoon climate areas, which provide suitable climatic conditions.
